# Early Dynamics of Zinc-Based Nanofertilizer Absorption in Plants of *Glycine max* L. (Fabaceae): Short-Term Ultrastructural and Functional Changes and Subcellular Localization

**DOI:** 10.3390/plants15142226

**Published:** 2026-07-21

**Authors:** Emilio de Castro Miguel, Sergimar Kennedy de Paiva Pinheiro, Alex Natã Bazzanezi, Karlos Eduardo Pianoski, Bruno Sousa Araújo, Barbara M. Santos, Michele A. S. Nobrega, Gilberto J. Arruda, Etenaldo F. Santiago, Montcharles S. Pontes, Thaiz Batista Azevedo Rangel Miguel

**Affiliations:** 1 Biomaterials Laboratory, Department of Metallurgical Engineering and Materials and Analytical Center, Campus do Pici, Federal University of Ceará, Fortaleza 60440-554, CE, Brazil; emiliomiguel@ufc.br (E.d.C.M.); kennedybiomat@ufc.br (S.K.d.P.P.); 2 PrimeAgro, R. Alberto Dalcanale, 3825—Vila Industrial, Toledo 85905-415, PR, Brazil; alexbazzanezi@primeagro.com.br (A.N.B.); karlospianoski@primeagro.com.br (K.E.P.); 3 Department of Physics, Center of Sciences, Federal University of Ceará, Fortaleza 60400-900, CE, Brazil; s.araujobruno@fisica.ufc.br; 4 Natural Resources Program, Center for Natural Resources Study (CERNA), Mato Grosso do Sul State University (UEMS), Dourados 79540-000, MS, Brazil; 04895157113@academicos.uems.br (B.M.S.); arruda@uems.br (G.J.A.); felipe@uems.br (E.F.S.); 5 Environmental Management and Technology Program, Federal University of Rondonópolis (UFR), Rondonópolis 78736-900, MT, Brazil; nobrega_michele@yahoo.com.br; 6 Optics and Photonics Group, Institute of Physics, Federal University of Mato Grosso do Sul (UFMS), Campo Grande 79070-900, MS, Brazil; montcharles.pontes@gmail.com

**Keywords:** foliar application, *Glycine max* L., nanofertilizers, biological barriers, Zinc (Zn^2+^)

## Abstract

This study investigated the initial absorption dynamics and ultrastructural responses of soybean leaf tissue to foliar application of zinc-based (Zn^2+^) nanofertilizer, using brightfield light microscopy, electron scanning and transmission microscopy (SEM and TEM), and Raman spectroscopy. Anatomically, no relevant structural changes were detected in the roots or stems in any of the treatments. However, leaves treated with ionic Zn^2+^ (T1) exhibited thickening of the palisade parenchyma with Zn^2+^ accumulation in the adaxial epidermis. Samples treated with T2 and T3 revealed epidermal precipitates and intracellular deposits within leaf cells, as evidenced by brightfield optical microscopy and Raman spectroscopy. These aggregates were absorbed and subsequently dissolved, being no longer observed at the 30 min time point. Ultrastructural analysis confirmed the presence of nanofertilizers within the vacuoles and cytoplasm, suggesting absorption and translocation of Zn^2+^, reinforcing the ability of nano-enabled materials to overcome biological barriers. Our results indicate that zinc-based (Zn^2+^) nanofertilizers can alter cellular features without evident short-term phytotoxicity. Theoretically, this study provides mechanistic insights into nanoparticle dissolution dynamics, biological barrier penetration, and subcellular compartmentalization pathways in crop plants. From an applied perspective, the rapid absorption (<30 min) and preferential organelle targeting suggest potential for precision nutrient delivery systems, though long-term and efficacy evaluations are required before agricultural deployment can be recommended.

## 1. Introduction

One of the main challenges faced by humanity is feeding a continuously growing population [1]. It is estimated that the global population will reach 9.8 billion by 2050 and up to 11.2 billion by 2100, leading to a sharp increase in the consumption of global natural resources [2]. To meet this demand, food production will need to increase by 5.1 billion tons before 2050 [2]. This will place enormous pressure on agricultural ecosystems, as they are the primary sources of food. Moreover, such demand may have negative environmental impacts, given that agricultural production consumes large amounts of water and energy [3], in addition to the widespread use of fertilizers and agricultural chemicals. The current demand for food production has been accompanied by an increase in the use of fertilizers, pesticides, and agrochemical inputs such as herbicides, insecticides, and fungicides. Chemical crop-protection agents (agrochemicals) are widely used compounds around the world and can be described as the main contributors to the increase in agricultural food productivity in recent decades, which has benefited agribusiness [4].

The excessive application of agrochemicals can undermine the circular economy in agriculture by generating waste and pollution for natural ecosystems [5]. A key factor in eliminating waste and pollution is the rationalization of pesticide and fertilizer usage. This goal can be more easily achieved through the application of nanotechnological tools. In this regard, nanotechnology offers environmentally friendly methods to enhance agricultural production [6], as engineered nanomaterials exhibit unique physicochemical properties. Due to these characteristics, their use has increased significantly in recent years in various technological processes and products [7]. Among these technological products, nanofertilizers and nanopesticides stand out, as they improve plant efficiency in nutrient absorption and enable targeted delivery of chemical cargo [8]. Such products minimize significant production losses and substantially reduce the use of fertilizers and pesticides [9].

Nanofertilizers are nutrients encapsulated, coated with nanomaterials, or a mineral element on a nanometric scale designed to overcome biological barriers and enable controlled release followed by their subsequent slow diffusion into the soil or foliage tissues [10]. The use of nanoscale fertilizers can help minimize nutrient loss through leaching/runoff and reduce their rapid degradation and volatility. Consequently, they improve nutrient quality and soil fertility, thereby promoting long-term crop productivity. Furthermore, due to their high surface-to-volume ratio and high penetration capacity, nanofertilizers can be a suitable alternative to conventional chemical fertilizers [11]. Currently, the market offers a range of nanofertilizers in testing or field application, as reviewed by [12]].

Recent studies have demonstrated that nanoparticle uptake by plant cells is governed by cell wall porosity (typically <10–20 nm) [13], although this barrier can be modified through nanoparticle-induced remodeling. ZnO nanoparticles specifically induce cell wall restructuring via the upregulation of lignin synthesis genes, arabinogalactan proteins, and pectin degradation pathways. This contrasts with AgNPs, which primarily cause cell wall loosening, and CuO NPs, which induce physical damage and biochemical rupture of cellulose microfibrils [13].

The organic and inorganic components of the soil can modify the effects of applied nanostructured materials, depending on their chemical nature and their interactions with the soil. Aggregation may occur, making them less mobile in porous materials as their size increases. Thus, the amount of organic matter in the soil, the surrounding environment, and the chemical properties of the nanofertilizers can either increase or reduce the mobility of the nanoparticles [13]. These nanofertilizers, depending on their concentration, are non-toxic and less harmful to humans and the environment than conventional fertilizers [14]. Additionally, they increase soil fertility, crop yield, and quality, while minimizing costs and optimizing profits [15].

Despite the growing interest in nanofertilizers, critical knowledge gaps remain regarding the following [16]: (i) the dynamics of nanoparticle absorption and dissolution at the leaf surface; (ii) subcellular localization and translocation pathways across biological barriers; (iii) ultrastructural responses of organelles, particularly chloroplasts, to nanofertilizer exposure; and (iv) real-time tracking of Zn species transformation from application to cellular internalization.

In this study, the potential anatomical and ultrastructural responses of soybean seedlings (*Glycine max* L.—Fabaceae-soy) under zinc-based nanofertilizer (Zn^2+^) application and their subcellular localization were investigated in detail. These cellular aspects could be key to the implementation of nano-enabled sustainable and precision agricultural strategies. This study addresses these gaps by investigating the short-term temporal dynamics of foliar-applied Zn-based nanofertilizer absorption in soybean seedlings using a multi-technique approach combining brightfield microscopy, SEM, TEM, and Raman spectroscopy. We specifically focus on: (1) characterizing the kinetics of nanoparticle dissolution and uptake; (2) identifying the subcellular compartments involved in Zn accumulation; and (3) assessing early ultrastructural responses that may indicate cellular adaptation or stress.

## 2. Materials and Methods

### 2.1. Materials

Soybean seeds were placed in polyethylene pots containing Plantimax-type agricultural substrate. The resulting seedlings were grown in a seedling nursery, specifically a growth greenhouse, under controlled light, temperature, and humidity conditions, with automated daily irrigation (three times per day) via micro-sprinklers. The treatments applied were a negative control (T0) (no application), zinc chloride (ZnCl_2_) (Treatment 1—T1), commercial fertilizer (Underi^®^, PrimeAgro, Toledo, Brazil) (Treatment 2—T2), and nanoformulation PR 112 zinc oxide nanoparticles (Treatment 3—T3). Both the Underi^®^ fertilizer and the PR 112 formulation contain zinc in their composition. Applications were performed using the concentration recommended by the manufacturer, adjusting the Zn^2+^ concentration to the same value for all applied treatments. Spraying was standardized at 50 mL of solution (300 mg/L) per individual plant at stages V4 or V5. Samples for microscopy analyses were collected at 0, 15, 30, and 60 min after solution application (T0, T1, T2, and T3).

The hydrodynamic diameter and polydispersity index (PDI) of the particles were determined by dynamic light scattering (DLS) using a Zetasizer Nano ZS (Malvern Panalytical, Malvern, UK) at 25 °C with a 173° backscattering angle, with nanoparticles dispersed in deionized water (See Table S1 in Supplementary Materials).

### 2.2. Microscopy Analysis

For microscopy analyses, plant fragments were fixed at room temperature in an aqueous solution containing 2.5% glutaraldehyde, 4.0% formaldehyde, and 0.1 M sodium cacodylate buffer at pH 7.2 for 24 h. Subsequently, the samples were washed three times for 45 min each in 0.05 or 0.1 M sodium cacodylate buffer and post-fixed for two hours in 1% osmium tetroxide in the same buffer at room temperature. After three additional 45 min washes in the same buffer, the samples were subjected to dehydration through a graded acetone series.

### 2.3. Brightfield Optical Microscopy

Plant fragments were fixed and dehydrated as previously described. Subsequently, for embedding, acetone was gradually replaced by EMbed 812 resin (Epoxy)—Pensilvânia (PA), USA. The resin was polymerized at 60 °C for 48 h. Then, 0.5 µm thick sections were obtained using a Leica UC7 ultramicrotome (Vienna, Austria) with glass knives. The sections were placed on slides and stained with 1.0% toluidine blue. Slide analysis and imaging were performed using a brightfield optical microscope (Primo Star-Zeiss—Suzhou, China) equipped with a digital capture system.

### 2.4. Scanning Electron Microscopy (SEM)

For the study of micromorphology of plant regions (stem and leaf), scanning electron microscopy (SEM) was used. Material fragments were fixed and dehydrated as previously described, followed by critical point drying. Subsequently, the fragments were mounted on aluminum stubs and sputter-coated with 20 nm of gold (Quorum QT150ES, East Sussex, UK). Observations and imaging were conducted using a scanning electron microscope (Quanta 450FEG-ThermoFisher, Brno, Czechia) with an accelerating voltage of 20 kV. Secondary electron images and backscattered electron images were obtained.

### 2.5. Transmission Electron Microscopy (TEM)

To determine the ultrastructure of cells, morphology, and the localization of nanoparticles (NPs), transmission electron microscopy (TEM) was used. For this purpose, the material was fixed, dehydrated, and embedded in resin as previously described. After resin polymerization, ultrathin sections (70 nm thick) were obtained. These sections were collected on 300-mesh copper grids and contrasted with 5% uranyl acetate for 40 min and 1% lead citrate for 5 min [16]. The material was analyzed using a TEM (JEM-1400 Plus, Akishima, Japan) with an accelerating voltage of 120 kV.

### 2.6. Raman Spectroscopy

Plant samples were mounted on microscope slides as previously described. Raman spectroscopy measurements were performed using 632.8 nm (1.96 eV) laser excitation, focused on the samples with a 10× objective lens (1.4 NA) coupled to an upright optical microscope model BX41 (Tokyo, Japan) configured in a backscattering geometry. The Raman-scattered radiation was analyzed using a Jobin–Yvon LABRAM-HR spectrometer (Villeneuve-d’Ascq, France) equipped with a charge-coupled device operating with a 1800 lines/mm diffraction grating. Spectra of the samples decorated with NPs were obtained with an accumulation time of 30 s, and Raman mapping was performed over an area of 100 × 100 μm, covered by a 64 × 64 pixel grid. Raman spectra of the control samples were acquired with an accumulation time of 25 s, and Raman spectroscopy mapping was conducted over an area of 100 × 100 μm, covered by a 32 × 32 pixel grid. Contributions from any sample degradation to the spectra are minimized due to the summation of signals [17]. The analysis of the Raman spectral dataset was carried out using a direct classical least squares (DCLS) modeling procedure.

### 2.7. AI-Assisted Graphical Abstract Generation

The graphical abstract was generated using the generative artificial intelligence tool Gemini 3.5 Flash (Google LLC, Mountain View, CA, USA) in March 2026. The image was created using prompts describing make a graphical abstract for the MS. The generated image was subsequently curated to ensure scientific accuracy. The authors retain full responsibility for the integrity and accuracy of the presented visual content.

## 3. Results

### 3.1. Anatomical and Micromorphological Analyses

Microscopy analyses were performed from the beginning to the end of the experiment over a total period of 60 min. Root, stem, and leaf tissues were carefully examined to identify anatomical and morphological variations among treatments. Transverse root sections from treatment T0 displayed a typical organization in primary growth, with elongated epidermal cells possessing thin walls, large parenchyma cells with thin walls, and a narrow cytoplasm adjacent to the cell wall. Below this layer, the endodermis and pericycle layers were observed, followed by the vascular bundle. Slightly below the collar region, transverse sections revealed the secondary root structure, showing the vascular cambium, phloem, and secondary xylem. Throughout the entire analysis period, no significant anatomical changes were observed; thus, the structure remained unaltered at all analyzed time points.

Careful observation of the roots of plants exposed to the fertilizers, using both scanning electron microscopy and optical microscopy, revealed an anatomy similar to that of the control plants. No evidence of aggregates suggestive of particle accumulation was detected in any of the tissues. Sections of roots in primary growth showed elongated epidermal cells with thin walls, large parenchymatic cells with thin walls, and narrow cytoplasm adjacent to the cell walls. The endodermis and pericycle were also observed, followed by the vascular bundle. Cross-sections of the organ in secondary growth revealed a typical structure, displaying vascular cambium, phloem, and secondary xylem. No significant anatomical changes were observed during the analysis.

The stems of the studied plants exhibited only a secondary growth structure, with no primary structure observed in any of the sections. This is due to the age of the plants and the sampling location. The experiment was conducted 90 days after sowing, and samples were collected from the median region of the plant. Histological sections revealed primary structure transitioning to a secondary structure in samples from the control experiment. These sections were characterized by the presence of an epidermis with more than one layer, a sclerenchyma ring surrounding almost the entire organ, vascular cambium, primary and secondary phloem, and secondary xylem predominating over primary xylem. Pith parenchyma cells were also observed. This structural pattern was maintained throughout all analyzed time points.

Observation of the histological sections from samples exposed to foliar fertilizer treatments revealed a structure, under all conditions and time points, similar to that of the control. Notably, in treatment T1 (Zn^2+^), the stem in secondary growth exhibited a periderm. No anatomical modifications were observed in relation to the control in any treatment or at any time point after exposure to the fertilizers.

**Figure 1:**
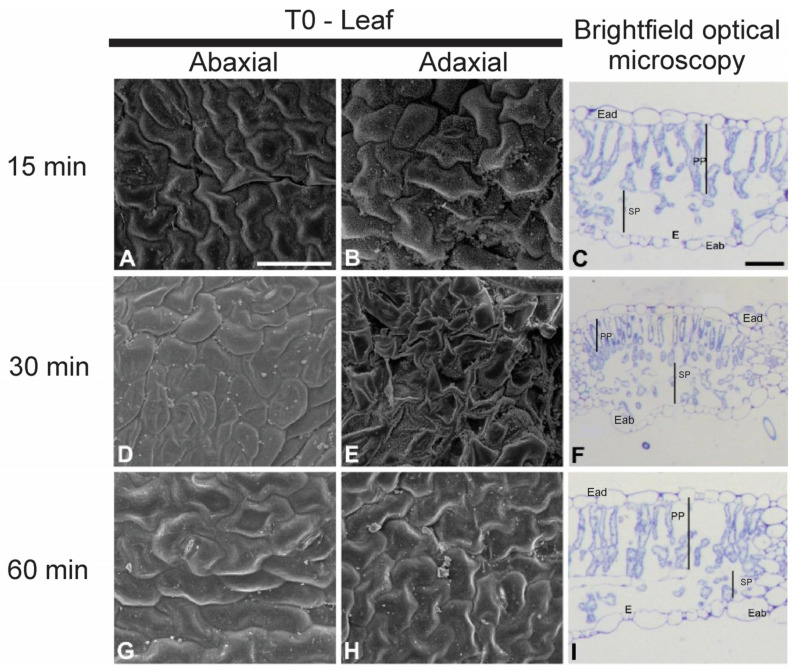
**Scanning electron microscopy (SEM) and brightfield optical microscopy of soybean leaves *(Glycine max* L.—Fabaceae) in the control treatment (T0):** (**A**–**C**) 15 min after application of distilled water; (**D**–**F**) 30 min after application of distilled water; (**G**–**I**) 60 min after application of the fertilizer. The micromorphological and anatomical structures were repeated at the three analyzed times. Ead—adaxial epidermis; PP—palisade parenchyma; SP—spongy parenchyma; E—stomata; Eab—abaxial epidermis. Scale bars: (**A**,**B**,**D**,**E**,**G**,**H**) 100 µm; (**C**,**F**,**I**) 50 µm.

SEM analysis of the leaf samples from the control experiment (T0) revealed epidermal cells with sinuously shaped anticlinal walls on both adaxial and abaxial surfaces, concave periclinal walls, and the presence of epicuticular wax. Stomata were observed on the abaxial surface, along with sparse trichomes. This structure was consistent at 15, 30, and 60 min of the experiment. Transverse sections of the leaf observed under optical microscopy showed a uniseriate adaxial epidermis, one to two layers of palisade parenchyma with cytoplasm containing numerous chloroplasts, 5–8 layers of cells in the spongy parenchyma, and a uniseriate abaxial epidermis with stomata and scattered tector trichomes (Figure 1). A similar organization was observed at 15, 30, and 60 min of the experiment (Figure 1).

**Figure 2:**
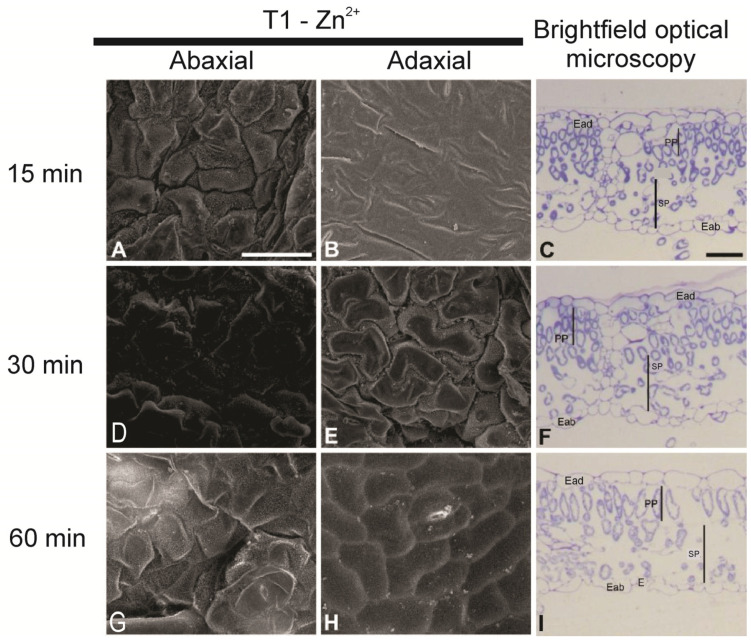
**Scanning electron microscopy (SEM)** and brightfield **optical** microscopy of soybean (*Glycine max* L.—Fabaceae) leaves under Zn^2+^ treatment (T1): (**A**–**C**) 15 min after distilled water application; (**D**–**F**) 30 min after distilled water application; (**G**–**I**) 60 min after fertilizer application. The micromorphological and anatomical structures were consistent across all three time points; however, they differed from the control in the number of palisade parenchyma and spongy parenchyma cells. Ead—adaxial epidermis; PP—palisade parenchyma; SP—spongy parenchyma; E—stomata; Eab—abaxial epidermis. Scale bars: (**A**,**B**,**D**,**E**,**G**,**H**) 100 µm; (**C**,**F**,**I**) 50 µm.

The sample treated with Zn^2+^ (T1) exhibited a leaf surface very similar to that of the control, preserving all features previously observed under SEM. However, transverse sections of the leaves examined under optical microscopy revealed an increase in palisade parenchyma cells, which now consist of three to four layers and display dense cytoplasm with numerous chloroplasts. The palisade parenchyma retains the same characteristics described for the control (Figure 2). This pattern was consistent at 15, 30, and 60 min after fertilizer application (Figure 2).

**Figure 3:**
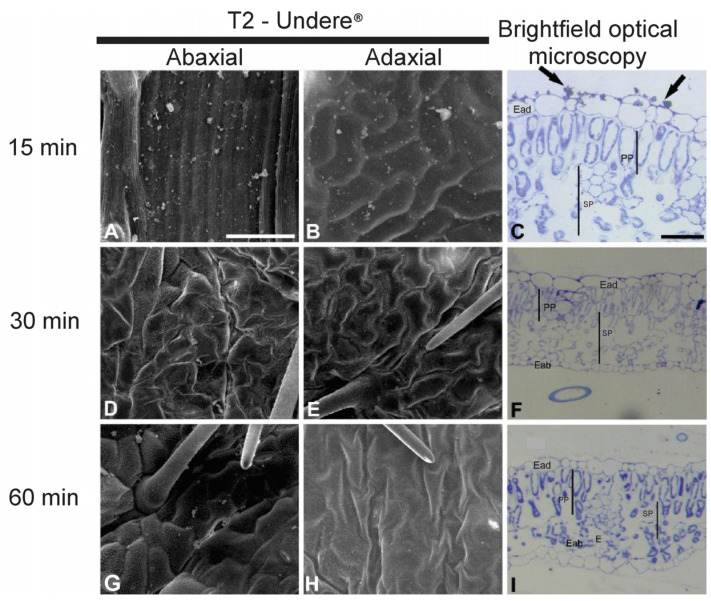
**Scanning electron microscopy (SEM)** and brightfield **optical** microscopy of soybean (*Glycine max* L.—Fabaceae) leaves under Underi^®^ treatment (T2): (**A**–**C**) 15 min after distilled water application; (**D**–**F**) 30 min after distilled water application; (**G**–**I**) 60 min after fertilizer application. The micromorphological and anatomical structures were consistent across all three time points; however, they differed from the control in the number of palisade parenchyma and spongy parenchyma cells. In this sample, precipitates on the adaxial epidermis were observed at the 15 min time point. Ead—adaxial epidermis; PP—palisade parenchyma; SP—Spongy parenchyma; E—stomata; Eab—abaxial epidermis. Scale bars: (**A**,**B**,**D**,**E**,**G**,**H**) 100 µm; (**C**,**F**,**I**) 50 µm.

**Figure 4:**
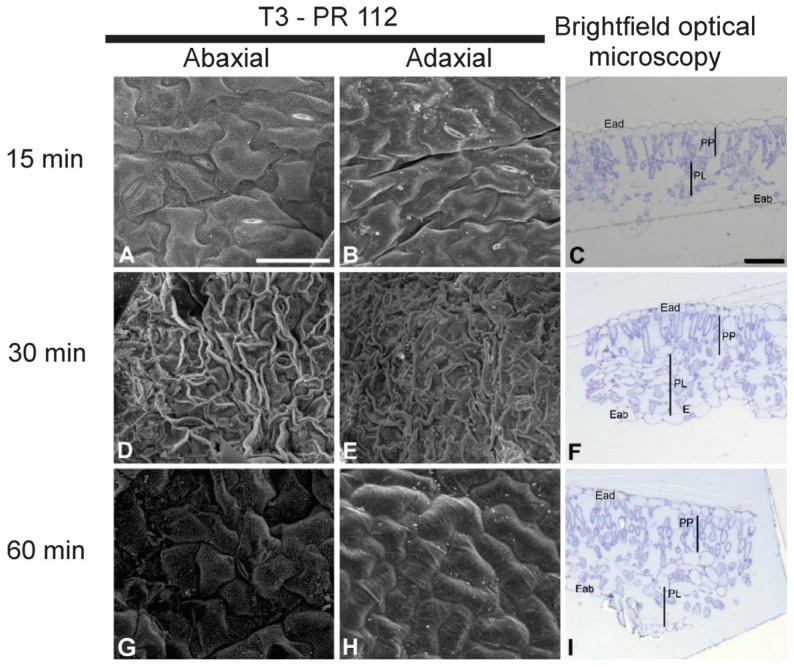
**Scanning electron microscopy (SEM)** and brightfield optical microscopy of soybean leaves (*Glycine max* L.—Fabaceae) under PR 112 (T3) treatment: (**A**–**C**) 15 min after distilled water application; (**D**–**F**) 30 min after distilled water application; (**G**–**I**) 60 min after fertilizer application. The micromorphological and anatomical structures were consistent across the three time points analyzed; however, they differed from the control in the number of palisade and spongy parenchyma cells. In this treatment, precipitates on the adaxial epidermis were visible at 15 min. Ead—adaxial epidermis; PP—palisade parenchyma; SP—spongy parenchyma; E—stomata; Eab—abaxial epidermis. Scale bars: (**A**,**B**,**D**,**E**,**G**,**H**) 100 µm; (**C**,**F**,**I**) 50 µm.

SEM analysis of the plants exposed to the fertilizers: commercial (T2) and nanoformulation (T3) revealed abaxial and adaxial leaf surfaces with the same features described for the other treatments. However, transverse sections of the leaves observed under brightfield optical microscopy showed notable anatomical differences. Although the anatomy varied similarly to that described for treatment T1, in these two treatments, at 15 min after commercial fertilizer (T2) application, it was possible to detect the accumulation of precipitates on the adaxial epidermis (Figure 3C). At 30 and 60 min after fertilizer application, these structures were no longer observed (Figure 3F,I). It was also observed that the application of nanofertilizer PR 112 (T3) at 15 min resulted in the accumulation of material within the adaxial epidermal cells (Figure 4C). At 30 and 60 min after nanofertilizer (T3) application, these structures were no longer observed (Figure 4F,I).

### 3.2. Raman Spectroscopy Analysis

**Figure 5:**
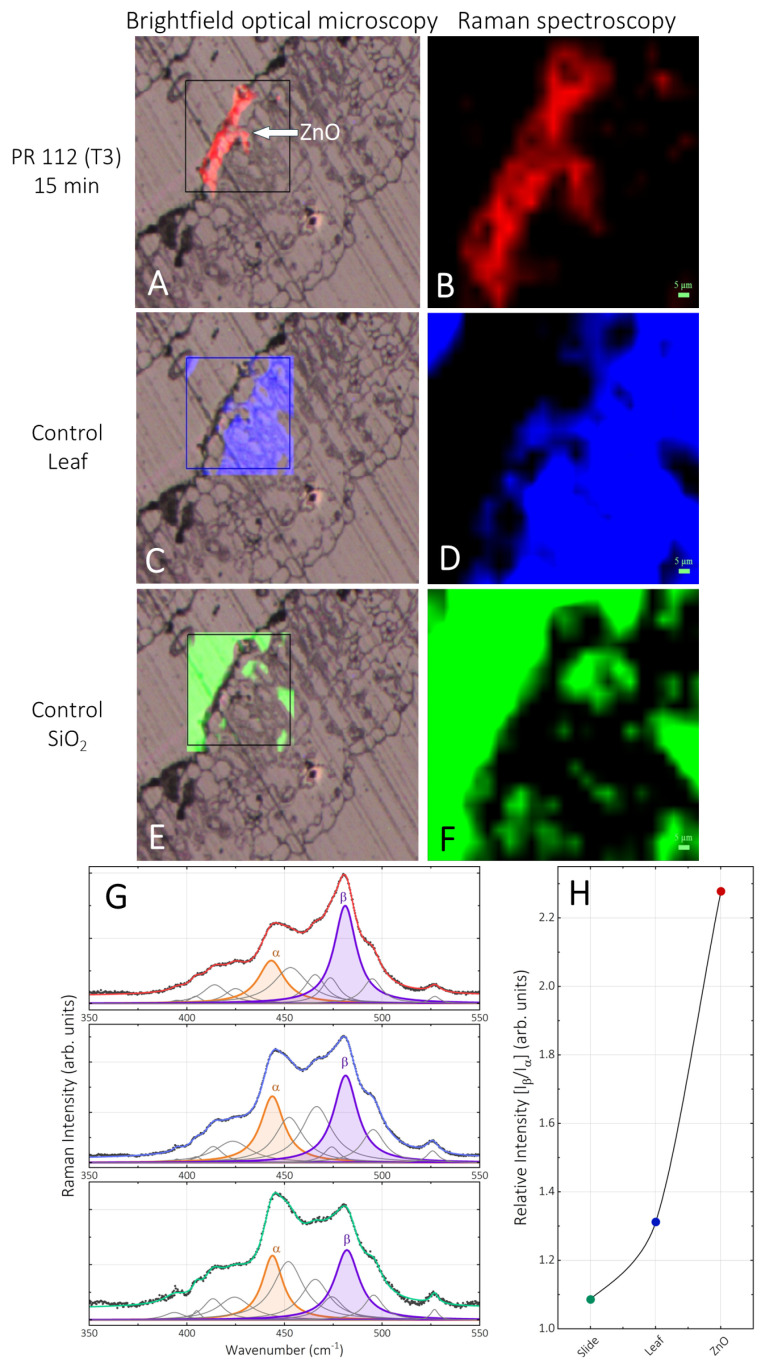
*Brightfield optical microscopy and Raman spectroscopy mapping of ZnO nanoparticles in leaf tissues:* (**A**,**B**) Leaf section exposed to ZnO nanoparticles (Pr 112). Brightfield image (**A**) shows red-colored agglomerates (arrow) corresponding to ZnO. The Raman map (**B**) highlights their spatial distribution in red, based on characteristic vibrational bands. (**C**,**D**) Control leaf without nanoparticle exposure. Brightfield image (**C**) and Raman map (**D**) show the absence of a ZnO signal, with blue indicating the native spectral profile of leaf tissue. (**E**,**F**) Control with SiO_2_. Brightfield image (**E**) and Raman map (**F**) reveal green-colored regions corresponding to the SiO_2_ spectral signature. (**G**) Representative Raman spectra (350–550 cm^−1^) for ZnO (top), leaf control (middle), and SiO_2_ control (bottom). Peaks α (~435 cm^−1^) and β (~510 cm^−1^) correspond to characteristic vibrational modes of the ZnO structure, with fitted components for spectral deconvolution. (**H**) Relative intensity ratio (Iβ/Iα) showing marked increase for ZnO compared with controls, demonstrating the method capability for selective and quantitative detection of NPs in plant tissues.

**Figure 6:**
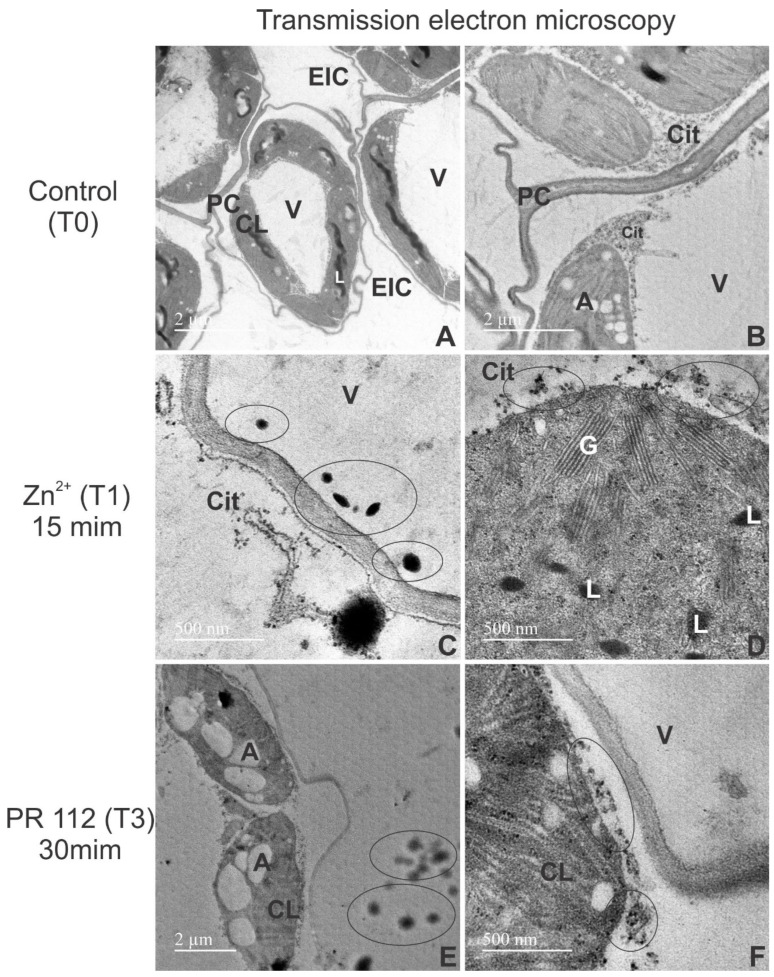
Transmission electron microscopy (TEM) of leaf tissue cells in the control and Zn^2+^ and PR 112 treatments: (**A**,**B**) **Control (T0):** Intact cellular organization showing epidermal (EIC) and palisade (PC) cells, vacuoles (V), chloroplasts (CL), lipid bodies (L), starch grain in the chloroplast (A), and cytoplasm (Cit) without electron-dense deposits. (**C**,**D**) Zn^2+^ treatment (T1, 15 min). Presence of electron-dense granules (ellipses) localized in the cytoplasm (Cit) and near vacuolar membranes (V), as well as in the vicinity of grana (G) and lipid bodies (L) within chloroplasts. (**E**,**F**) PR 112 treatment (T3, 30 min). Accumulation of electron-dense aggregates (ellipses) in chloroplasts (CL), with deposits also detected adjacent to vacuolar membranes (V). PC—cell wall; V—vacuole; CL—chloroplast; EIC—intercellular space; Cyt—cytoplasm; A—starch grain in the chloroplast; L—lipid inclusions in the cytoplasm; G—grana; Ellipse—nanoparticle inclusions.

Figure 5 shows the correlation between brightfield optical microscopy and Raman spectroscopy for treatment T3 (nanofertilizer) to identify and characterize ZnO nanoparticles in leaf tissue, comparing the treated and control samples. Figure 5A displays the accumulation of ZnO particles (red) in the adaxial epidermis, highlighted by the arrow indicating ZnO. The area enclosed by the square corresponds to the region analyzed by Raman spectroscopy (Figure 5B), where the spectral intensity characteristic of ZnO is represented in red, indicating a strong concentration of the nanomaterial in the selected region. Figure 6C shows the preserved leaf structure, without the presence of particles, with the area of interest marked in blue. In the Raman spectroscopy mapping (Figure 5D), blue regions indicate the spectral response characteristic of leaf tissue without any ZnO-associated signal, confirming the absence of the nanomaterial. In Figure 6E, the cellular distribution appears similar to the control leaf, but with the analysis area highlighted in green. The Raman spectroscopy mapping (Figure 5F) shows a green signal corresponding to the characteristic bands of SiO_2_, confirming the presence of the reference material in the tissue.

The three graphs shown in Figure 5G present the spectral signatures for each experimental condition (ZnO, control leaf, and SiO_2_) within the 350–550 cm^−1^ range. The α and β peaks, indicated on the fitted curves, correspond to vibrational modes characteristic of each material, enabling a clear distinction between ZnO (with more intense and shifted peaks) and the controls. The scatter plot (Figure 5H) depicts a progressive increase in the β/α intensity ratio, being lowest for SiO_2_ (green point), intermediate for the leaf (blue point), and highest for ZnO (red point), demonstrating the sensitivity of the Raman microspectroscopy for detecting and discriminating NPs within a biological matrix.

### 3.3. Cell Ultrastructure

The ultrastructural analysis of leaf cells from the control experiment revealed a thin cell wall with poorly defined middle lamellae, a large central vacuole, and an abundance of chloroplasts occupying most of the cytoplasm. The chloroplasts exhibited structures strongly suggestive of starch grains and lipid inclusions. Large intercellular spaces were also observed (Figure 6A,B).

Although no precipitates were detected in any tissue of the plants exposed to Zn^2+^ treatment (T1) under light microscopy, transmission electron microscopy revealed small precipitates located in both vacuoles and cytoplasm. Precipitates could not be clearly visualized inside the chloroplasts (Figure 6B,C). The chloroplasts displayed a typical structure, with no alterations in any compartment. Leaves from plants exposed to the nanofertilizer (T3) treatment exhibited precipitates in the cytoplasm and vacuole, as well as modifications in the chloroplasts, which presented larger grana with fewer stacks and large starch grains, thereby altering the typical chloroplast structure (Figure 6E,F).

## 4. Discussion

### 4.1. Anatomical and Micromorphological Analyses

The Zn concentration applied in all treatments was standardized at 300 mg/L in order to compare the treatments. The maintenance of typical anatomical structures (roots, stems, and leaves) across all treatments indicates that the applied fertilizers did not cause detectable morphological or structural damage, not even in the histological sections or SEM analyses. This behavior is consistent with the results from foliar Zn applications in other species, where no anatomical alterations are observed, but rather, micronutrient enrichment occurs [18,19].

Notably, the accumulation of precipitates in the adaxial leaf epidermis was observed in T2 (15 min) and T3 (15 min) in sections analyzed by brightfield optical microscopy. This phenomenon likely reflects the precipitation of elements such as zinc carbonate or phosphates, common in formulations with poorly soluble minerals [20]. The absence of these precipitates at subsequent time points (30 and 60 min) suggests reabsorption or redistribution following application, which aligns with the dynamics of foliar feeding: rapid initial absorption followed by translocation within the tissue [21,22]. Importantly, no phytotoxicity symptoms were observed in any treatment, including the chloride-based treatment (T1), as evidenced by anatomical and ultrastructural analyses.

### 4.2. Raman Spectroscopy Analysis

The combined analyses of brightfield optical microscopy and Raman spectroscopy mapping in treatment T3 (PR 112) allowed for the unequivocal identification and distribution of ZnO particles in the adaxial leaf epidermis. In Figure 5A,B, the intense signal in the red-highlighted region on the leaf surface corresponds to the characteristic spectral signature of ZnO in the 350–550 cm^−1^ range, matching vibrational modes previously described for this oxide [23]. This spatial correlation between optical observation and Raman mapping reinforces the robustness of the method for localized detection of nanomaterials. The preservation of leaf structure in particle-free samples (Figure 5C,D) and the absence of vibrational peaks associated with ZnO indicate that the material is not present in the control, preventing false positives. The reference sample with SiO_2_ (Figure 5E,F) served as an internal spectral control, displaying characteristic peaks within the same spectral range but with distinct shifts and intensities, consistent with the vibrational behavior of quartz and amorphous silica [24].

The overlaid spectral signatures (Figure 6G) demonstrate that ZnO exhibits α and β peaks that are more intense and slightly shifted compared to the controls, which can be attributed to the differences in crystal structure, particle size, and interactions with the biological matrix [25]. This spectral differentiation is critical for distinguishing ZnO from materials with partially overlapping bands, such as carbonates or silicates naturally present in leaves. The scatter plot (Figure 5H) highlights differences in the relative intensity ratio β/α: minimal for SiO_2_, intermediate for leaf tissue, and maximal for ZnO, revealing the sensitivity of the spectral index as a quantitative marker. This type of quantitative approach has previously been suggested to monitor the accumulation of metallic NPs in plant tissues, including ZnO, using intensity ratios between vibrational modes as reliable discriminators [25].

### 4.3. Ultrastructural Analysis

After entry into the leaf tissue, NPs and their aggregates can be absorbed by the cell wall if their dimensions are smaller than its pore diameter [26]. Cell wall porosity is estimated to be <10 nm [27], with an upper limit of <20 nm [28]. However, universal values for size exclusion limits cannot be established due to structural heterogeneity among species and cell types. Moreover, evidence suggests that NPs may induce cell wall remodeling by activating the genes responsible for lignin synthesis and degradation [29], as well as arabinogalactan proteins [28], hemicelluloses, and pectins [30]. Exposure to NPs may result in increased cell wall pore size, thereby facilitating the entry of these particles [31]. Studies in *Lactuca sativa* demonstrated that silver nanoparticles (AgNPs) induce cell wall loosening [17], whereas copper oxide nanoparticles (CuO NPs) cause physical damage and promote biochemical rupture of cell walls, weakening the connections between cellulose microfibrils [32]. These findings suggest that the initial cellular response to NPs may involve cell wall remodeling.

At the ultrastructural level, leaf cells from the control (T0) exhibited normal features, such as chloroplasts with starch grains, vacuoles, and abundant intercellular space. In T1 (Zn^2+^), despite the absence of visible precipitates in SEM, transmission electron microscopy (TEM) detected precipitates in the vacuoles and cytoplasm of *Glycine max* leaves, suggesting Zn^2+^ internalization. This aligns with studies indicating that small ZnO particles can penetrate tissues [31], even without accumulation being detectable by SEM [32]. In the nanoformulation PR 112 treatment (T3), in addition to precipitates, chloroplasts displayed alterations such as more widely spaced grana and larger starch grains, possibly reflecting altered metabolism due to the presence of micronutrients (e.g., Zn, Ca, P) or adjuvants in the formulation. Previous studies indicate that chloroplast modifications may be linked to changes in photoassimilate balance and carbohydrate storage [33]. The preferential vacuolar and chloroplast localization of Zn (Figure 6) suggests active compartmentalization, potentially for detoxification (vacuole) and metabolic utilization (chloroplast). The chloroplast alterations observed (increased grana spacing, starch accumulation) may reflect Zn incorporation into photosynthetic enzymes or antioxidant responses.

### 4.4. Mechanistic Insights: From Foliar Application to Subcellular Localization

Our temporal analysis reveals a multi-step process: (i) Precipitates on adaxial epidermis (Figure 3C and Figure 4C) indicate initial particle aggregation. Penetration likely occurs via stomata and cuticle pores, facilitated by small particle sizes and surfactants in formulations. (ii) Precipitate disappearance coincides with Zn detection in mesophyll (TEM, Figure 6). This suggests a rapid dissolution to Zn^2+^ ions at pH ~5–6 (leaf apoplast), and/or direct nanoparticle endocytosis. The observed Zn localization in vacuoles (pH ~5) may promote further ZnO dissolution, releasing Zn^2+^ for metabolic use. (iii) Zn translocation to chloroplasts (Figure 6E,F) parallels chloroplast structural changes: (i) increased grana spacing may reflect thylakoid membrane reorganization; (ii) starch accumulation suggests altered carbon partitioning, potentially due to Zn activation of ADP-glucose pyrophosphorylase or inhibition of starch degradation enzymes. These ultrastructural responses differ from phytotoxicity markers (e.g., chloroplast swelling, membrane disruption, cristae disorganization in mitochondria), supporting the conclusion of biocompatibility within the tested timeframe.

## 5. Conclusions

In summary, from an anatomical and morphological perspective, analyses using light microscopy, scanning electron microscopy (SEM), and transmission electron microscopy (TEM) revealed the maintenance of typical tissue structures in roots, stems, and leaves, with no evidence of toxicity or structural reorganization in plant tissues. The exception was ultrastructural alterations in chloroplasts of leaves treated with the PR 112 nanofertilizer (T3), which exhibited thicker grana, increased starch accumulation, and intracellular precipitates, suggesting possible modulation of photosynthetic activity. The presence of nanofertilizer was primarily detected in the cytoplasm and vacuoles, with no visible accumulation in external tissues. Treatments T3 and T4 showed aggregates on the surface and inside leaf cells. These aggregates were subsequently absorbed and dissolved, as they were not observed at the 30 min time point. The rapid dissolution kinetics (<30 min) documented here inform formulation strategies. Coatings or matrices that control ZnO dissolution rates could enable sustained release, matching plant uptake capacity while minimizing losses. Preferential targeting of chloroplasts, the primary site of photosynthetic Zn utilization, suggests that nanofertilizers can deliver nutrients directly to metabolically active organelles, potentially reducing application rates compared to conventional fertilizers. These findings reinforce the potential of nanofertilizers as an efficient alternative for nutrient supply, promoting biomass gains without compromising plant anatomical integrity. Additionally, Raman microspectroscopy demonstrates a higher sensitivity for sensing nanofertilizer within a biological matrix.

## Data Availability

The original contributions presented in this study are included in the article. Further inquiries can be directed to the corresponding author.

## Supplementary Materials

The following supporting information can be downloaded at: https://www.mdpi.com/article/10.3390/plants15142226/s1. Based on the data analysis from your DLS measurements, here are the suggested caption and methodology sentence. Table S1. Hydrodynamic diameter and polydispersity index of PR-112 nanoparticles. Mean hydrodynamic diameter (Z-Average) and polydispersity index (PDI) values obtained from dynamic light scattering measurements. Data represent mean ± standard deviation (n = 3).
