# Supplementary material for: Early Dynamics of Zinc-Based Nanofertilizer Absorption in Plants of Glycine max L. (Fabaceae): Short-Term Ultrastructural and Functional Changes and Subcellular Localization
Source: Plants (Basel). 2026 Jul 21;15(14):2226. doi: 10.3390/plants15142226 (PMC13415319; doi:10.3390/plants15142226)
Supplement: Supplementary file 1 [file plants-15-02226-s001.zip › plants-4099476-supplementary.pdf]

Early Dynamics of Zinc-Based Nanofertilizer Absorption in Plants of *Glycine max* L. (Fabaceae): Short-Term Ultrastructural and Functional Changes and Subcellular Localization

Emilio de Castro Miguel [1], Sergimar Kennedy de Paiva Pinheiro [1], Alex Natã Bazzanezi [2], Karlos Eduardo Pianoski [2], Bruno Sousa Araújo [3], Barbara M. Santos [4], Michele A. S. Nobrega [5], Gilberto J. Arruda [4], Etenaldo F. Santiago [4], Montcharles S. Pontes [6] and Thaiz Batista Azevedo Rangel Miguel [1,*]

[1] Biomaterials Laboratory, Department of Metallurgical Engineering and Materials and Analytical Center, Campus do Pici, Federal University of Ceará, Fortaleza 60440-554, CE, Brazil;
emiliomiguel@ufc.br (E.C.M.); kennedybiomat@ufc.br (S.K.d.P.P.)

[2] PrimeAgro, R. Alberto Dalcanale, 3825—Vila Industrial, Toledo, 85905-415, PR, Brazil;
alexbazzanezi@primeagro.com.br (A.N.B.);
karlospianoski@primeagro.com.br (K.E.P.)

[3] Department of Physics, Center of Sciences, Federal University of Ceará, Fortaleza 60400-900, CE, Brazil;
s.araujobruno@fisica.ufc.br

[4] Natural Resources Program, Center for Natural Resources Study (CERNA), Mato Grosso do Sul State
University (UEMS), Dourados, 79540-000 MS, Brazil;
04895157113@academicos.uems.br (B.M.S.);
arruda@uems.br (G.J.A.); felipe@uems.br (E.F.S.)

[5] Environmental Management and Technology Program, Federal University of Rondonópolis (UFR),
Rondonópolis 78736-900, MT, Brazil; nobrega_michele@yahoo.com.br

[6] Optics and Photonics Group, Institute of Physics, Federal University of Mato Grosso do Sul (UFMS),
Campo Grande 79070-900, MS, Brazil; montcharles.pontes@gmail.com

* Correspondence: thaizrangel@gmail.com

Table S1. Based on the data analysis from your DLS measurements, here are the suggested caption and methodology sentence: Table S1 Caption Table S1. Hydrodynamic diameter and polydispersity index of PR-112 nanoparticles. Mean hydrodynamic diameter (Z-Average) and polydispersity index (PDI) values obtained from dynamic light scattering measurements. Data represent mean ± standard deviation (n=3)

	Nanofertilizer PR112
PDI	0.211 ± 0.009
Diameter (nm)	278.30 ± 5.38
